# Supplementary material for: From sequence to enzyme mechanism using multi-label machine learning
Source: BMC Bioinformatics. 2014 May 19;15:150. doi: 10.1186/1471-2105-15-150 (PMC4229970; doi:10.1186/1471-2105-15-150)
Supplement: Additional file 2 — Java code of ml2db. Additional file ml2db_code.tar.gz contains the Java source code to run the multi-label machine learning experiments and save the results to database. The code’s Javadoc is included. [file 1471-2105-15-150-S2.zip › additional file 2/ml2db/ml2db/doc/mulan/evaluation/loss/class-use/RankingLossFunction.html]

Uses of Interface mulan.evaluation.loss.RankingLossFunction


JavaScript is disabled on your browser.


- Overview
- Package
- Class
- Use
- Tree
- Deprecated
- Index
- Help

- Prev
- Next

- Frames
- No Frames

- All Classes

## Uses of Interface mulan.evaluation.loss.RankingLossFunction

- Packages that use RankingLossFunction

  | Package | Description |
  | --- | --- |
  |  |  |
  | --- | --- |
  | mulan.classifier.neural |  |
  | mulan.evaluation.loss |  |
  | mulan.evaluation.measure |  |
- - ### Uses of RankingLossFunction in mulan.classifier.neural

    Constructors in mulan.classifier.neural with parameters of type RankingLossFunction

    Constructor and Description

    `MMPLearner(RankingLossFunction lossMeasure,
    MMPUpdateRuleType modelUpdateRule)`

    Creates a new instance of `MMPLearner`.

    `MMPLearner(RankingLossFunction lossMeasure,
    MMPUpdateRuleType modelUpdateRule,
    long randomnessSeed)`

    Creates a new instance of `MMPLearner`.

    `MMPMaxUpdateRule(java.util.List<Neuron> perceptrons,
    RankingLossFunction lossMeasure)`

    Creates a new instance of `MMPMaxUpdateRule`.

    `MMPRandomizedUpdateRule(java.util.List<Neuron> perceptrons,
    RankingLossFunction lossMeasure)`

    Creates a new instance of `MMPRandomizedUpdateRule`.

    `MMPUniformUpdateRule(java.util.List<Neuron> perceptrons,
    RankingLossFunction lossMeasure)`

    Creates a new instance of `MMPUniformUpdateRule`.

    `MMPUpdateRuleBase(java.util.List<Neuron> perceptrons,
    RankingLossFunction loss)`

    Creates a new instance of `MMPUpdateRuleBase`.
  - ### Uses of RankingLossFunction in mulan.evaluation.loss

    Classes in mulan.evaluation.loss that implement RankingLossFunction

    | Modifier and Type | Class and Description |
    | --- | --- |
    |  |  |
    | --- | --- |
    | `class` | `ErrorSetSize` Implementation of the ErrorSetSize loss function, which computes the size of the error set. |
    | `class` | `IsError` Implementation of the IsError loss function, which is simply the indicator of whether the induced ranking is perfect or not. |
    | `class` | `OneError` Implementation of the one-error loss function. |
    | `class` | `OneMinusAveragePrecision` Implementation of the average precision as loss function. |
    | `class` | `RankingLoss` Implementation of the "ranking loss" ranking loss function. |
    | `class` | `RankingLossFunctionBase` Base class for ranking loss functions |
  - ### Uses of RankingLossFunction in mulan.evaluation.measure

    Constructors in mulan.evaluation.measure with parameters of type RankingLossFunction

    Constructor and Description

    `LossBasedRankingMeasureBase(RankingLossFunction aLoss)`

    Creates a loss-based ranking measure

- Overview
- Package
- Class
- Use
- Tree
- Deprecated
- Index
- Help

- Prev
- Next

- Frames
- No Frames

- All Classes
